# An Approach of Vulnerability Testing for Third-Party Component Based on Condition and Parameter Mutation

**DOI:** 10.1155/2013/609254

**Published:** 2013-09-08

**Authors:** Jinfu Chen, Jiamei Chen, Yongzhao Zhan, Weihe Chen, Rubing Huang

**Affiliations:** ^1^School of Computer Science and Tele. Engineering, Jiangsu University, Zhenjiang 212013, China; ^2^School of Computer Science and Tech., Huazhong University of Science and Technology, Wuhan 430074, China

## Abstract

The research on component vulnerability testing is critical. In this paper, an approach of vulnerability testing is proposed based on condition mutation and parameter mutation in order to effectively detect the explicit vulnerabilities of third-party components. To start with, the Pre-condition Mutation Algorithm (*PCMA*) is presented to generate mutants set of the pre-condition and test cases are generated based on these mutants. Then, the Single Parameter Mutated Values (*SPMV*) procedure is addressed to generate parameter values based on mutation operators of parameter specification. These values are then taken as the input of the Test Case Generation Algorithm based on the Parameter Constraint (*TCGPC*), which is addressed to generate test case set violating the parameter constraint. The explicit vulnerabilities can be detected by the vulnerability detecting algorithm based on the test cases of condition and parameter mutation. The experiments show that our approach can detect explicit vulnerability faults of third-party components. Furthermore, the proposed approach can detect more vulnerability faults than other related approaches such as condition coverage methods, fuzzy testing method and boundary value method.

## 1. Introduction

With the development of component technologies, the number of the applications of the third-party components is increasing in some safety-critical software such as medical software and bank software. Over the past 30 years, the research mainly has focused on functionality testing, which does its best to find faults in developing and implementation of components. However, vulnerability testing of components, which detects component flaws threatening the security of the computer system by violating security requirements including memory leak and buffer overflow, has been ignored in the current component development, especially in the development of third party components. Since source codes of third party components are unavailable and third party components are highly independent, white-box testing technologies cannot be successfully applied, which leads to the challenges and difficulties for testing the vulnerability of third party components. In addition, current research on component security testing is rare, which mainly focuses on security characterization, security assessment, component deployment and wrapper testing method, security testing based on fault injection, formal methods, Jabeen and Jaffar-Ur Rehman [1] proposed security requirement specification for enhancing testability of component security, which provided specifications from the perspectives such as resources allocation, environment deployment, and method invocation. However, it did not figure out specific testing approach. Bertolino and Polini [2] addressed a framework for component deployment testing, which added a spy class in the tested component to collect and compare running state of the tested component and the resources allocation. In case of related running status and environment variables violating security requirement specification, we can conclude that security vulnerabilities exist. Haddox et al. [3] presented a wrapper testing method, which wrapped tested component. Extra input and output interfaces are added for testing the component in the wrapper, and the data are allowed to flow into and out the component at the public wrapping interface level. The wrapper method can analyze third party components based on requirement specification in the theory. However, a theory model was given in the approach proposed by Bertolino and M. Haddox, whose feasibility is not verified by some effective experiments. In addition, Chen et al. [4] addressed a software security testing approach based on fault injection, which, to some extent, could detect explicit security vulnerabilities of components. But its testing process is complex, and the testing efficiency is not very ideal without considering the effect of interface parameter constraint and method precondition. The drawbacks of proposed methods are mainly lacking specific effective experimental approaches to verify the efficiency of the proposed methods. In order to address these drawbacks, a testing approach is presented based on testing method sequences. Testing method sequences have higher statement and branch coverage quality, which lead to better testing efficiency [5]. Therefore, considering the characteristics of explicit exceptions and the notion of specification mutation [6–8], this paper proposes an approach using condition mutation and parameter mutation based on method sequences. Since security vulnerabilities of most software are often caused by errors in judgment statements and conditional expressions, condition mutation method is presented. Firstly, the precondition is extracted from the requirement specification, and then test cases are generated, which satisfy and violate the precondition expression. Based on these test cases, whether security vulnerabilities exist or not is judged according to the postcondition expression. In the parameter mutation method, the corresponding mutation operators are firstly selected, according to a parameter type to generate test values. Then the test cases are generated based on value and relation constraint extracted from the requirement specification. Finally, the security exceptions will be detected by using component vulnerability detecting algorithm. This paper not only proposes a component vulnerability testing approach but also figures out the framework of the vulnerability testing approach. Some experiments are conducted to verify the feasibility of proposed approach.

The remainder of this paper is organized as follows. The vulnerability testing framework is described in the next section. Condition mutation testing algorithm is presented in Section 3 and parameter mutation testing algorithm is addressed in Section 4. Some experiments are conducted to verify our approach in Section 5. In the end, the conclusions are drawn in Section 6.

## 2. Vulnerability Testing Framework

**Figure 1:**
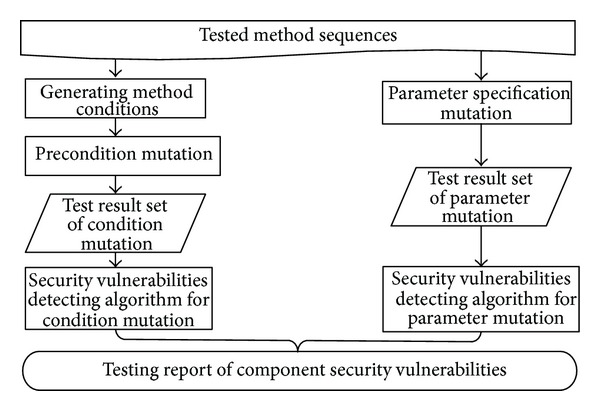
The framework of vulnerability testing.

In this section, a vulnerability testing framework will be described. A testing approach of condition and parameter mutation was presented based on component requirement specification, which is the main part of the framework. The vulnerability testing framework proposed in this paper is shown in Figure 1. In order to accurately describe the framework, several definitions are firstly given as follows.

**Definition 1**

Precondition (Prc) is a series of constraint conditions, which must be *true* before the method can be invoked.

**Definition 2**

Postcondition (Poc) is the condition which should be *true* after a method is invoked and decides the correctness of the operations performed. Postcondition can be expressed with such information as return value, the output result, parameter value, and environment variables.

**Definition 3**

Condition mutation operator, namely, relational operator reference fault operator (RRF), can convert the relational operator in a simple relational expression to reverse operator. The detailed information is shown in Table 1.

**Definition 4**

Requirement specification (RSF) of component security is described by XML format according to some schema, which is provided by developers or obtained through analyzing function description and IDL information by component users. Referring to requirement specification in the literature [9], method precondition, method postcondition, value, and relation constraint for method parameters are added to RSF.

**Definition 5**

Method sequences are feasible execution sequences that can be generated by data mining technology [10].

Based on the above definitions, vulnerability testing framework is described as follows. On the one hand, the precondition and postcondition of each method are extracted from RSF, and test data that meet precondition are generated. Then RRF mutation operator is applied to mutate precondition to generate precondition mutants, and test data that violate precondition are generated as method input based on precondition mutants. Finally method sequences are executed to detect whether vulnerabilities exist in the component by vulnerabilities detecting algorithm for condition mutation. On the other hand, parameter mutation generates test data that easily trigger component exceptions through using related mutation operators according to the parameter type. Then combinational testing method is applied to reduce the number of test cases, and test cases are selected by value and relation constrains. In the end, the method sequences are run and whether vulnerabilities exist will be judged by corresponding vulnerabilities detecting algorithm.

## 3. Condition Mutation Testing Algorithm

In this section, condition mutation testing algorithm is addressed. Component security vulnerabilities are often caused by wrong judgment statements and condition expressions. Incorrect relational operators usually lead the method to execute a different branch so that the method returns mistaken result. Condition mutation aims to test the relational expression in method precondition. Test cases that meet and violate precondition statement are generated. Combined with postcondition, test cases are input into method sequence to verify whether the vulnerabilities exist in judgment statement. Some related definitions are firstly given as follows before specific algorithms are described.

**Definition 6**

Precondition (Prc) consists of relational expressions and logical operators. In boolean logic, a boolean formula can be represented in the disjunctive normal form (DNF) which means that a boolean formula is in DNF if it is a disjunction of *cubes*, each of which is a conjunction of literals [11]. Therefore, Prc can be represented in DNF, namely, Exp_11_&&Exp_12_ … &&Exp_1*s*_||⋯||Exp_*m*1_&&Exp_*m*2_…&&Exp_*mt*_. A relational expression Exp_*ij*_ is regarded as a literal in boolean logic, and Exp_11_&&Exp_12_ … &&Exp_1*s*_ is a cube which is a conjunction of *s* relational expressions.

Method precondition and postcondition (see Definitions 1 and 2) are the expressions connecting parameters, environment variables (properties) with relational operators, logic operators, and arithmetic operators. For example, a banking withdraw method: *void withdraw *(int *a*), *a* is the withdrawing amount, *b* is new balance after the method is invoked, and *b*1 is the balance before the method is invoked, and then postcondition should be *a* + *b* = = *b*1.

**Definition 7**

Constraint equation set: Prc is short for the precondition in DNF, Prc = Exp_11_&&Exp_12_ … &&Exp_1*s*_||⋯||Exp_*m*1_&&Exp_*m*2_…&&Exp_*mt*_, the corresponding *m* constraint equation sets are expressed as follows:

(1) $$\begin{array}{c} \{\begin{array}{c} \text{Ex}{\text{p}}_{11}\\ \text{Ex}{\text{p}}_{12}\\ \vdots \\ \text{Ex}{\text{p}}_{1s} \end{array}\ldots \{\begin{array}{c} \text{Ex}{\text{p}}_{m1}\\ \text{Ex}{\text{p}}_{m2}\\ \vdots \\ \text{Ex}{\text{p}}_{mt}. \end{array} \end{array}$$

The relational expression Exp_*ij*_: *f*(*x*_1_, *x*_2_,…, *x*_*n*_)  ◊ 0,  ◊ is a relational operator, and *x*_*i*_ is a variable of the relational expression, so a constraint equation set is expressed as follows:

(2) $$\begin{array}{c} \{\begin{array}{c} f_{1}\left(x_{1},x_{2},\ldots ,x_{n}\right)⋄0\\ \ldots \\ f_{k}\left(x_{1},x_{2},\ldots ,x_{n}\right)⋄0. \end{array} \end{array}$$

**Definition 8**

If an equation includes only one variable, then the equation is simple, otherwise it is complex.

The subalgorithms on condition mutation are presented as follows.

*(1) Test Case Generation Approach Based on Constraint Equation Set (TCES). *A precondition can be expressed as a constraint equation set or several equation sets, and *TCES* is designed for solving these sets to assign certain value to each variable in the set. Finally the solutions of equation sets are merged. The solution procedure of the relational equation set is described as follows [12].

**Step 1**

Define the initial domain for a variable *x*_*i*_ according to the simple equation of *x*_*i*_, and the initial domain of other variables that do not appear in simple equations is (−*∞*, +*∞*). After definition, all simple equations are removed from the set.

**Step 2**

Variable *x*_*c*_ is selected as current variable that appears most frequently in complex equations or whose domain is the narrowest. Randomly select a value from the domain of *x*_*c*_, and assign the value to *x*_*c*_.

**Step 3**

Substitute the value of *x*_*c*_ into complex equations.

**Step 4**

If simple equations appear in the set after *x*_*c*_ is assigned, then, according to these simple equations, redefine the domain of variables that appear in simple ones. If the subset of two domains is empty, backtrack algorithm will be called.

**Step 5**

Repeat the above process until all variables are assigned.

There are several shortcomings in the above five steps. The backtrack algorithm in this approach is very time-consuming, and it restricts the efficiency of the algorithm if the equation set has no solution. Thus a criterion is designed for judging whether an equation set has solutions to avoid many backtracks to insoluble equation set. The criterion is shown as follows. In each equation, variables are moved to the left of the relational operator and constants are moved to the right. Then the algorithm can detect whether there is a group of equations, whose left expression sum is equal to zero, but right expression sum is not equal to zero. If the above situation appears, the equation set is insoluble.

It is supposed that there are *m* equation sets, these equation sets totally include *v* variables, and each set averagely has *k* equations. The order of the average complexity of *TCES* is *O*(*m* · *v* · *k*). Precondition mutation algorithm is illustrated as in Algorithm 1.

(2) *Precondition Mutation Algorithm (PCMA)*. *PCMA* algorithm is designed to generate all expressions that make precondition *false.* Prc is supposed to have *m* subitems. Firstly, *MSA*(*E*_0_) procedure is applied to obtain all mutants of the first subitem, and theses mutants are traversed, if one of them, for example, *t* and the mutant *s* from *MSA*(*E*_*i*_), do not own mutually exclusive relation expressions by *IsExclusive* procedure, then *t*&&*s* is merged into *T*. After traverse is finished, the above procedure is repeated with *T* and *E*_2_ up to *E*_*m*_. For analyzing the complexity of *PCMA*, *MSA*(*E*_*i*_) is supposed averagely to have *k* mutants, and then the order of the complexity of algorithm is *O*(*m* · *k* ^2^).

*MSA*(*E*_*i*_) procedure uses RRF operator to generate mutants that make *E*_*i*_ *false*. *IsExclusive*  procedure is designed for judging whether two subterms have mutually exclusive relation expression. Two procedures are respectively described as Procedure 1 (*MSA*) and Procedure 2 (*IsExclusive*). 

*MSA*() procedure is designed to obtain all mutants of subitem *E*_*i*_. It is supposed that *E*_*i*_ has *n* relation expressions. Each expression generates several mutants using RRF operator. For example, the expression is *a* > *b* and its mutant set is {*a* = *b*, *a* < *b*}. The expression and corresponding mutants are seen as one term of “Cartesian AND” that is similar to “Cartesian Product,” whose operator is replaced by logic AND. The final result removes *E*_*i*_. *MSA*() at most generates 3^*n*^-1 mutants.

This procedure is defined to judge whether *E*_*i*_ and *E*_*j*_ are mutually exclusive. If *E*_*is*_ being from *E*_*i*_ and *E*_*jt*_ being from *E*_*j*_ are mutually exclusive, then *true* is returned. For instance, *E*_*i*_ = *a* > 0 & & *b* > 10,  *E*_*j*_ = *a* < 10 & & *b* = 3, *b* > 10 in *E*_*i*_, and *b* = 3 in *E*_*j*_ are mutually exclusive, therefore *E*_*i*_ and *E*_*j*_ are mutually exclusive. Vulnerability detecting algorithms based on condition mutation are described as follows.

*(3) Security Vulnerabilities Detection Algorithm Based on Condition Mutation*. The *SVDACM* algorithm is shown as Algorithm 2. This algorithm can successively test each method in the method sequence. If the method has a precondition, the *TCES* algorithm is invoked to generate legal data for running the method. In case of any exception is thrown or the postcondition is violated after the tested method is run, then security vulnerabilities exist. In the meantime, *PCMA* algorithm is invoked to mutate the precondition, and then test cases are generated based on *TCES* algorithm to violate precondition. If the method is successfully run, then the tested component is insecure. In addition, if the method has no precondition, test cases are generated by boundary value and fuzzy testing approach combined with postcondition to detect whether the method is correct.

For analyzing the time complexity of *SVDACM*, it is supposed that the method number of sequences is *p*, the average number of each sequence is *q*, precondition has *m* subitems, every subitem includes *k* expressions, and *v* variables are included. Since the order of the time of *TCES* is *O*(*m* · *v* · *k*) and that of *PCMA* is *O*(*m* · 3^2*k*^), the order of the time complexity of *SVDACM* is *O*(*p* · *q* · (*m* · *v* · *k* + *m* · 3^2*k*^)). 

## 4. Parameter Mutation Testing Algorithm

The purpose of parameter mutation is to generate the data set that can easily trigger underlying errors in the component method. Firstly, a series of values is generated through using all related mutation operators according to the parameter type. For numeric parameter, values assigned to the parameter which meet value constraint are selected so as to be input into the tested method. For a parameter of another type, values which violate value constraint are selected. Combinational testing method is used to reduce the number of test cases. Final test cases which violate relation constraint are selected to trigger security exception. If any exception is triggered in the methods or the postcondition is violated, it is demonstrated that the method is insecure and exceptional. Test cases and method information are saved to further find out the location of security exception in the tested method. Several definitions are given as follows related to specific algorithms.

**Definition 9**

Value constraint means that the parameter value is restricted in the certain scope. For example, *index* is the index of an array, and then value constraint of *index* is *index *≥0. For another example, *a* is denoted as an edge variable of a triangle, and then the constraint of *a* is *a* > 0.

**Definition 10**

Relation constraint means that constraint may exist between parameters, which is described as the expression that is prone to be mistaken or be omitted. For instance, a method whose function is to judge the type of a triangle has 3 parameters, that is, *a*, *b*, *c* for three edges of a triangle, and then a programmer possibly makes a mistake or omits the judgment statement of nontriangle. Thus, relation constraint of the method is such expression as *a* + *b* > *c* && *a* + *c* > *b* && *b* + *c* > *a*.

**Definition 11**

18 mutation operators related to parameters are proposed based on the literature [4] according to eight parameter types, namely, integer, char, float, Boolean, string, pointer, array, and structure. They are shown in Table 2.

In Table 2, AIV operator is designed for an array to generate irregular values. For example, the sequence of elements of the array is changed into ascending order, descending order, and disorder. The value of the element located into particular position is changed just as *a*[0] which stores the length of the array assigned to a negative number. The value of the element is set to certain value such as *minimum* ± 1, *maximum* ± 1, and normal value. The length of the array is changed. SIV operator is designed for a structure type parameter, which is used to mutate simple members of a structure. If the parameter type is *integer*, these operators including PSN, IPO, PFB, and IIV are used to generate mutation integer values. If the parameter type is *char*, these operators including PSN, IPO, PFB, and CIV are used to generate irregular values and change the value of a *char* parameter into mutated values. Similarly, PSN and FIV operators are used to mutate a parameter of type* float*, and PSN and BIV operators are used to mutate a parameter of type *Boolean*; PSN, RSV, LSV, FSV, DSV, USV, CSV, SSI, and CSS operators are applied for a *string* parameter to generate random string and other strings which can trigger security exceptions. PSN and PIV operators are conducted to make pointer parameter point to freed memory and the end of the allocated memory to trigger security exceptions. For an *array* parameter, PSN and AIV operators are used to generate mutated values. PSN and SIV operators are applied for a *structure* parameter to generate irregular values and special values which can trigger explicit exceptions for every member of the *structure*.

The test case generation algorithms based parameter constraint are described as follows.

(1) *Test Case Generation Based on Parameter Constraint (TCGPC).* Data set is generated by calling single parameter mutated values (*SPMV*) procedure corresponding parameter type. Since the size of this set is very large, combinational testing method is applied to reduce the size of test case set. Test cases which do not meet relation constraint are selected to trigger security exceptions. *TCGPC* algorithm is described as in Algorithm 3.

The main steps of *TCGPC* algorithm are illustrated as follows. Firstly, parameter values are generated by calling *SPMV* procedure corresponding parameter type for each parameter of the tested method. Then if the parameter type is numeric, values which meet value constraint are selected. Otherwise, values which do not meet value constraint are also selected. If the tested method includes only one parameter, then the corresponding result set is returned. If the number of parameters is two, then pairwising testing is applied. If the tested method includes no less than 3 parameters, then 3-tuple combinational testing method is used for generating combinational test cases. Finally, test cases which do not meet relation constraint are selected to trigger errors. For analyzing the time complexity of *TCGPC* algorithm, it is supposed that the tested method has *n* (*n* ≥ 3) parameters, and the number of the *i*th parameter value is denoted as *v*[*i*], these *v*[*i*] are obtained after *SPMV* procedure is called. In addition, *d* is denoted as *v*[0] (*v*[0] ≥ *v*[1] ≥ ⋯≥*v*[*n* − 1]). The order of the time complexity before combinational testing is *O*(*n* · *d*). Referring to the literature [13], the order of the time complexity of combinational testing is *O*(*d* ^*n*+3^ · *n* ^2^ + *d* ^4^ · *n* ^2^ · log⁡(*n*)), and then the order of the time complexity after combinational testing is *O*(*C*_*n*_^3^ · *d* ^3^). Thus the order of the time complexity of *TCGPC* is *O*(*C*_*n*_^3^ · *d* ^3^ + *d* ^*n*+3^ · *n* ^2^ + *d* ^4^ · *n* ^2^ · log⁡(*n*)).

(2) *Single Parameter Mutated Values (SPMV).* This procedure uses all related operators according to the parameter type to generate test cases. It is shown as in Procedure 3.

*SPMV* procedure generates parameter values for eight types of parameters using corresponding operators that are listed in Table 2. Vulnerability detecting algorithms based on parameter mutation are described as follows.

 (3) *Security Vulnerability Detecting Algorithm Based on Parameter Mutation*. The *SVDAPM* algorithm is shown as Algorithm 4. *SVDAPM* will scan each method of each sequence. If a method includes at least one parameter, *TCES* algorithm is invoked to obtain test cases, which are inputted into the method, then the tested method is run. If the actual result is different from exception result, then it is shown that the test case is effective. Furthermore, the testing result will be written into *PMR*. It is assumed that there are *p* sequences, and each sequence includes *m* methods, and then the order of the time complexity of *SVDAPM* algorithm is *O*(*p* · *m* · (*C*_*n*_^3^ · *d* ^3^ + *d* ^*n*+3^ · *n* ^2^ + *d* ^4^ · *n* ^2^ · log⁡(*n*)).

## 5. Experiments and Analyses

**Figure 2:**
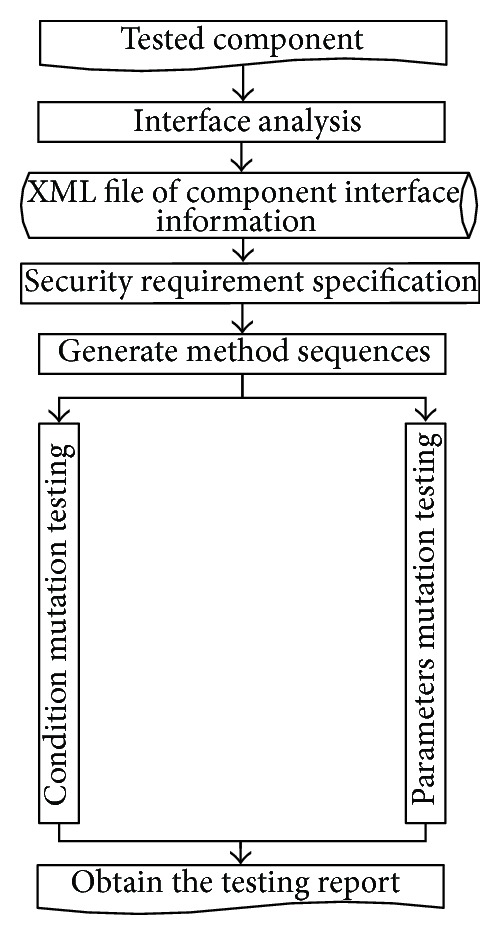
The testing process of condition and parameter mutation.

In order to verify the feasibility of the proposed approach and corresponding algorithms, some experiments are, respectively, conducted based on condition and parameter mutation approach. The experiments are performed in C# language based on common environment such as Windows XP, Visual Studio, NET 2008 development environment, PC with 2 GB memory, 2.93 GHz CPU, and 500 GB compatible hard disk. The complete testing process is described as follows. (1) Analyze the interface information of the third party component based on type library to obtain component interface information; (2) security requirement specification is defined according to component description and IDL information; (3) the precondition and postcondition of the tested method are extracted from specification, and method sequences are mutated; (4) value and relation constraints are extracted from specification, and method sequences are mutated; (5) vulnerability detecting algorithms are called, and vulnerability testing report is generated. The testing process is shown in Figure 2.

### 5.1. Experiment and Analysis of Condition Mutation Testing

In order to verify the feasibility of condition mutation approach, two components which exist in explicit vulnerabilities, that is, TestCondiDll1.dll and TestCondiDll2.dll, are tested in the experiment. The detail information of two components is shown in Table 3. TestCondiDll1.dll has 6 methods, and the number of code line is 63; TestCondiDll2.dll is composed of 7 methods, which includes 70 code lines. An RRF fault is injected into each method, and thus the first component has 6 faults injected and the second one has 7 faults injected.

The experimental result of TestCondiDll1 is shown in Table 4, which lists some information including method name, precondition of the method, mutated Prc using RRF operator, type-number of test cases that meet Prc(type-number of detecting the fault), and type-number of test cases that violate Prc (type-number of detecting the fault). For example, subtract method has 5 types of test cases that meet Prc which are {*a* > *b*&&*b* > *c*,  and *a* > *b* && *b* = *c*, *a* > *b* && *b* < *c*, *a* < *b* && *b* > *c*, *a* = *b* && *b* > *c*}, among which, *a* < *b* && *b* > *c* can detect the fault. {*a* = *b* && *b* = *c*, *a* = *b* && *b* < *c*, *a* < *b* && *b* = *c*, *a* < *b* && *b* < *c*} are 4 types of test cases that violate Prc, among which, {*a* = *b*&&*b* = *c*, *a* = *b*&&*b* < *c*, *a* < *b* && *b* < *c*} can distinguish Prc and Prc′. It is shown from the table that type-number of test cases is related to the number of relational expressions and opening (closing) interval of a variable. The more relational expressions are, the larger the number of types is. In addition, there are more types if a variable has opening interval rather than closing interval. It is also shown that condition mutation can effectively detect faults caused by RRF operator. 

In addition, to verify and analyze the testing capability about detecting component explicit exception, condition mutation approach is compared with decision coverage, condition coverage and multiple condition coverage, by testing six methods of TestCondiDll1. The comparison result is shown in Table 5. Two test cases are obtained which, respectively, make Prc be *true* and *false* in decision coverage approach. Test cases are generated by making each relational expression of Prc be *true* and *false* in condition coverage approach. Multiple condition coverage requires test cases that cover all the conditions in a decision. By analyzing Table 5, we can see that the number of test cases that are generated by other 3 methods is the subset of that of the condition mutation. However, other 3 methods uncertainly can find all faults injected. Condition mutation approach generates most test cases, but it can find all faults caused by RRF operator. It is obvious that the condition mutation approach is effective.

### 5.2. Experiment and Analysis of Parameter Mutation Testing

The experiment is conducted for verifying the feasibility of parameter mutation. TestParam.dll is tested in the experiment. TestParam.dll has 7 methods, 85 code lines, and 7 faults injected. The detail information is shown in Table 6.

The experimental result is shown in Table 7 for TestParam.dll component. Table 7 shows some testing information such as name of a method, value constraint of corresponding parameter, relation constraint of parameters, time generating cases, number of all cases, number of cases that find faults, and detecting rate. It is obvious that our approach is effective.

**Figure 3:**
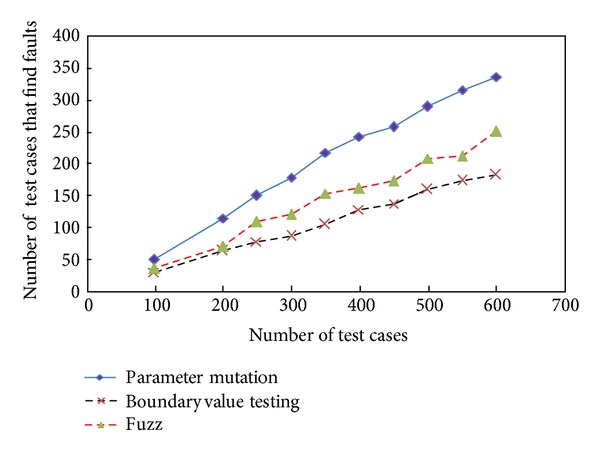
The comparison with fuzzy testing method and boundary value method.

In order to obverse the validity of parameter mutation, parameter mutation is compared with boundary value testing and fuzzy testing method. Boundary value testing means that test cases are designed by using variable values at their extreme points such as maximum (max), max⁡−1, minimum (min), min⁡+1, and nominal value (nom) [14]. Fuzzy testing is a security testing method which injects random input value into the parameters of a function in order to obtain an unexpected behavior and identify potential vulnerabilities [15, 16]. The comparison result is shown in Figure 3, from which, we can see that the more test cases generated are, the more effective cases are. The detecting efficiency of boundary value method is the lowest, that of fuzzy testing method is in the middle, and that of parameter mutation is the highest. With the number of test cases increasing, the advantage of parameter mutation tends to be more obvious.

## 6. Conclusions and Future Work

Since some detailed design information and source codes are unavailable in the third party component, it brings a large number of difficulties into component vulnerability testing. In this paper, the approach of vulnerability testing-based condition and parameter mutation is proposed according to the characteristics of explicit exceptions. The advantages and disadvantages of proposed approach are summarized as follows.

(1) Condition mutation approach addresses *TCES* algorithm to generate test cases that meet precondition and mutation *PCMA* algorithm to get several mutants. By combining these mutants with *TCES*, test cases that violate precondition are generated, and then component vulnerabilities can be detected by *SVDACM* algorithm. Parameter mutation approach adopts *TCGPC* algorithm to generate test data through using all related operators corresponding parameter type. In addition, test cases set becomes smaller when combinational testing method is used. Some test cases that violate relation constraint are selected. *SVDAPM* algorithm is applied to detect component vulnerabilities from the perspective of the parameter fault.

(2) Component security specification that is used in our approach is comprehensive, which not only records component information of methods and properties but also includes some detailed information such as method precondition, method postcondition, and parameter constraint. Condition and parameter mutation algorithms (*PCMA* and *SPMV*) are presented to generate mutated precondition and parameter value based on security testing framework. Vulnerabilities detecting algorithms (*SVDACM* and *SVDAPM*) are addressed to detect whether the component is secure or not. In the end, the experiments show that the proposed approach can detect some explicit exceptions and the proposed approach is feasible. Furthermore, the experiments also show that condition mutation method can detect more vulnerability faults than decision coverage, condition coverage, and multiple condition coverage methods. The parameter mutation method is also compared with fuzzy testing and boundary value methods, and the comparison result shows that the effectiveness of parameter mutation method is higher than the other two methods.

(3) However, the proposed approach could not obtain good testing result if the method of tested component did not have this information such as precondition, postcondition and parameter constraints. In addition, the approach in this paper is designed for detecting explicit exceptions; as a result, implicit exceptions of component cannot be effectively detected. In the future, state mutation approach for method sequences will be explored in detail according to characteristics of implicit security vulnerabilities. It is promising that some meaningful changes are made into method sequences to generate insecure or unreachable method sequences. These insecure sequences are executed, and then the executed result is observed by judging whether they are successfully run to detect implicit exceptions of the tested component.

## Figures and Tables

**Algorithm 1:**
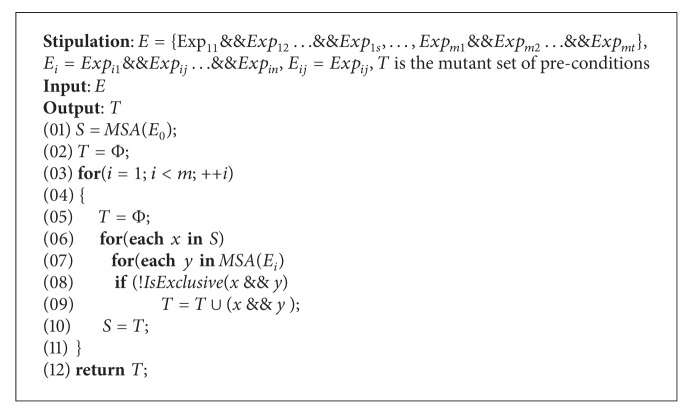
PCMA.

**Procedure 1:**
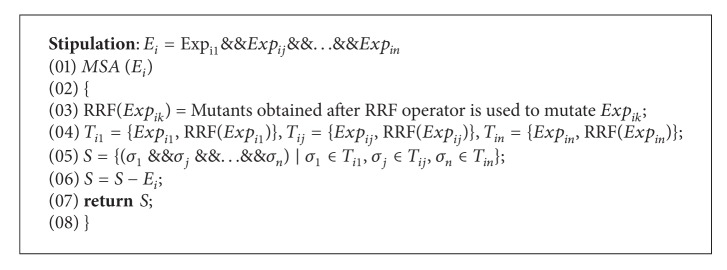
MSA(Mutation Sub-item Approach).

**Procedure 2:**
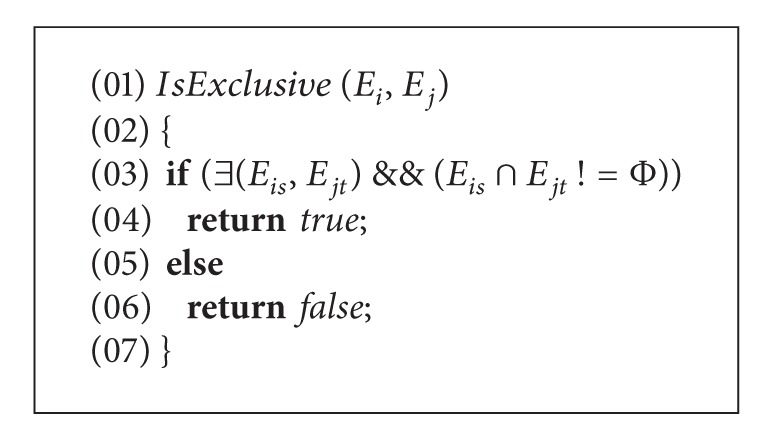
*IsExclusive*().

**Algorithm 2:**
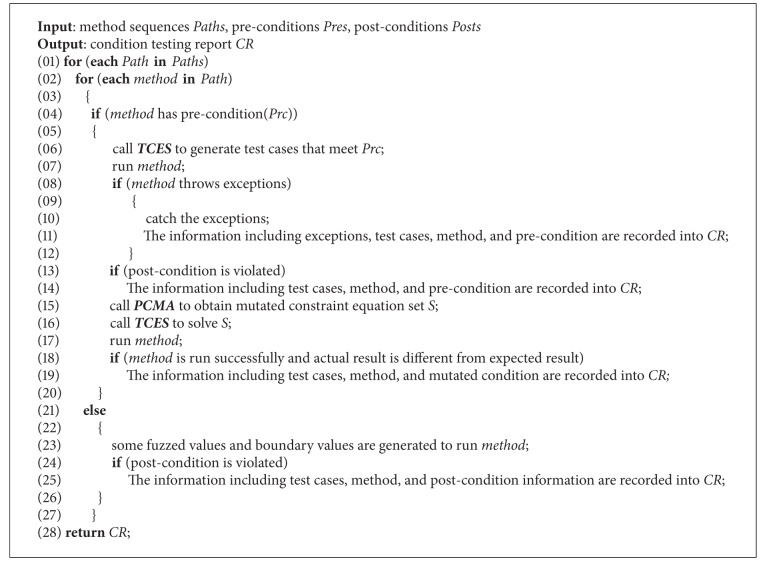
SVDACM.

**Algorithm 3:**
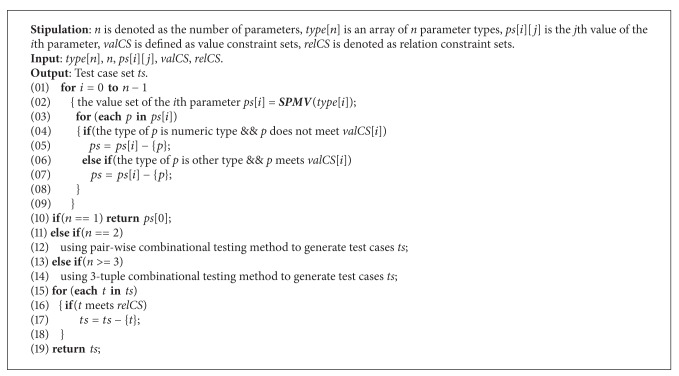
TCGPC.

**Procedure 3:**
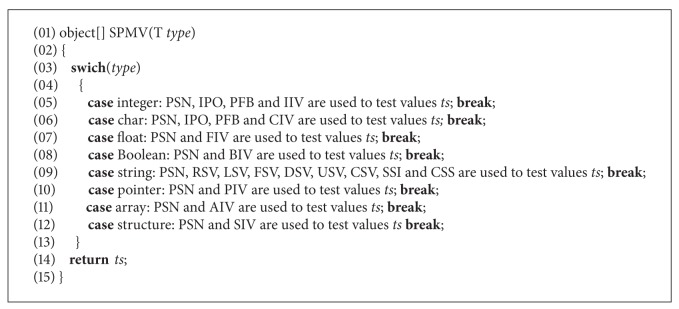
SPMV().

**Algorithm 4:**
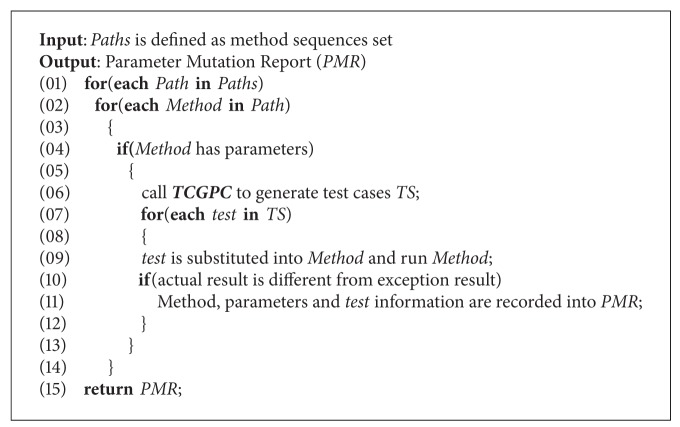
SVDAPM.

**Table 1:** RRF operator.

Before mutation	<		≤	>		≥	=		*≠*
After mutation	>	=	>	<	=	<	<	>	=

**Table 2:** Mutation operators of parameters of different types.

ID.	Operator	Brief description	Cases
01	PSN	Set the value of a nullable parameter to be Null	Set the value of a parameter whose value can be Null, such as, String a = *Null*; object b = *Null*;
02	IPO	Insert Parameter Operator into the value assigned to the parameter	Insert absolute value symbol or unary operator(++,− −,−,~) into the value assigned to the parameter
03	PFB	Parameter Flip Bit	Flip the value or flip the value of a bit
04	IIV	Integer Irregular Value	0, ±(1, 2^8^ − 1, 2^8^, 2^8^ + 1, 2^16^ − 1, 2^16^, 2^16^ + 1, 2^32^ − 1, 2^32^, 2^32^ + 1, 2^64^ − 1, 2^64^, 2^64^ + 1)
05	FIV	Float Irregular Value	0, ±(1, 3.402823*E* ^38^, 3.402823*E* ^38^ + 1, 3.402823*E* ^38^ − 1, 1.79769313486232*E* ^308^, 1.79769313486232*E* ^308^ + 1, 1.79769313486232*E* ^308^ − 1)
06	CIV	Char Irregular Value	‘A', ‘Z', null, ‘a', ‘z', ‘ ', ‘{ ', ‘(', ‘[', ‘∖n', ‘∖0', ‘∖s', ‘∖d'
07	BIV	Boolean Irregular Value	Correct, Incorrect, Tru, Fal, −1, 1
08	RSV	Random String Value	Escape character string“∖e∖n∖r∖d∖x∖s”, “∖xff∖xfe∖x00∖x01∖x42∖xb5∖nnnn∖h9cc...”
09	LSV	Long String Value	Generate String(int n) such as: “AAA……(256)”, “AAA……(1024)”, “AAA…(15000)”
10	FSV	Format the Value of String	“%n %n……(256 chars)”, “%s %s……(1024 chars)”
11	DSV	the Value of Directory String	“..”, “../”, “..//”, “/..//AAA…”
12	USV	URL and Value of File Path String	“http://dddddddeeeeerrttttt”, “C://sytem32//Notepad.exe”, “H:∖ABC∖killvirus.ese”, “D:∖AA.exeexe”
13	CSV	the Value of Command String	“;cmd.exe/c dir”, “del ∗.∗ /s”
14	SSI	SQL String Injection	“a or 1 = 1”, “delete”, “drop table users”
15	CSS	Cross Site Scripting	“<script>alert(document location);</script>”
16	PIV	Pointer Irregular Value	Null, −1, the pointer pointing to freed memory or to the end of the allocated memory
17	AIV	Array Irregular Value	Change the order of array elements into ascending, descending, or disorder order; change the value of array element to ± (maximum − 1, maximum + 1, maximum, minimum, minimum + 1, and minimum − 1); set the index of the array to (the length of array) ± 1
18	SIV	Structure Irregular Value	Set members of a structure to boundary values; Set every member to irregular values according to the member's type

**Table 3:** The information of two tested component.

ID.	Component name	Method number	Code line number	Number of faults injected
01	TestCondiDll1.dll	6	63	6
02	TestCondiDll2.dll	7	70	7

**Table 4:** Testing result for TestCondiDll1 using condition mutation.

Method name	Pre-condition(*Prc*)	Mutated *Prc*(*Prc*′)	Type-number of Test cases that meet *Prc *	Type-number of Test cases that violate *Prc *
*JudgeTriangle *	50 > a > 0&&50 > b > 0&&50 > c > 0	50 > a ≥ 0&&50 ≥ b > 0&&c > 50	1 (1)	124 (4)
*GetLargest *	(a > 100||b < 100)&&c > 0	(a < 100||b ≤ 100)&&c > 0	5 (1)	22 (3)
*Withdraw *	0 < a < 100	a ≥ 100	1 (1)	4 (2)
*Subtract *	a > b||b > c	a ≥ b||b < c	5 (1)	4 (3)
*Multiply *	(0 < a < 400||100 < b < 800)&&100 > c > 0	(0 < a ≤ 400||b = 800)&&100 > c > 0	9 (3)	116 (4)
*And *	0 < a < 400||100 < b < 800	0 < a < 400||b > 800	9 (4)	16 (4)

**Table 5:** The comparison with related testing approaches.

Testing approaches	Number of test cases	Number of faults found
Condition mutation	316	6
Decision coverage	12	0~6
Condition coverage	12	0~6
Multiple condition coverage	34	0~6

**Table 6:** The information of TestParam.dll.

ID.	Component name	Method number	Code line number	Number of faults injected
03	TestParam.dll	7	85	7

**Table 7:** The testing result of parameter mutation.

Method name	Value constraint	Relational constraint	Time for generating test cases	Number of all test cases	Number of test cases that find faults	detecting rate
*JudgeTriangle(int a, int b, int c) *	*a* > 0; *b* > 0; *c* > 0	*a* + *b* > *c*; *a* + *c* > *b*; *b* + *c* > *a*	218 ms	663	339	51.13%
*Add(int a, int b) *	*a* > −10; *b* > −10	*a* ≥ *b*	62 ms	60	15	25%
*Query(String s) *	—	—	422 ms	9	1	11.11%
*GetCharacterCount(String s) *	—	—	An infinite loop is caused by data overflow	9	1	11.11%
*IsAcuteTriangle(int a, int b, int c) *	a > 0; b > 0; c > 0	*a*∗*a* + *b*∗*b* = = *c*∗*c*	63 ms	964	543	56.33%
*CIsLargest(int a, int b, int c) *	a > 0; b > 0; c > 0	c ≥ a && c ≥ b	157 ms	615	112	18.21%
*IsQuotient(int a, int b, int c) *	b ≠ 0; a ≥ 0; c ≥ 0	c∗b = = *a*	250 ms	981	327	33.33%
